# Cation Dependence, pH Tolerance, and Dosage Requirement of a Bioflocculant Produced by *Bacillus* spp. UPMB13: Flocculation Performance Optimization through Kaolin Assays

**DOI:** 10.1100/2012/495659

**Published:** 2012-09-10

**Authors:** Zufarzaana Zulkeflee, Ahmad Zaharin Aris, Zulkifli H. Shamsuddin, Mohd Kamil Yusoff

**Affiliations:** ^1^Department of Environmental Sciences, Faculty of Environmental Studies, Universiti Putra Malaysia, 43400 UPM Serdang, Selangor, Malaysia; ^2^Environmental Forensics Research Centre, Faculty of Environmental Studies, Universiti Putra Malaysia, 43400 UPM Serdang, Selangor, Malaysia; ^3^Department of Land Management, Faculty of Agriculture, Universiti Putra Malaysia, 43400 UPM Serdang, Selangor, Malaysia; ^4^UPM Consultancy & Services Sdn. Bhd., Universiti Putra Malaysia, 43400 UPM Serdang, Selangor, Malaysia

## Abstract

A bioflocculant-producing bacterial strain with highly mucoid and ropy colony morphological characteristics identified as *Bacillus* spp. UPMB13 was found to be a potential bioflocculant-producing bacterium. The effect of cation dependency, pH tolerance and dosage requirement on flocculating ability of the strain was determined by flocculation assay with kaolin as the suspended particle. The flocculating activity was measured as optical density and by flocs formation. A synergistic effect was observed with the addition of monovalent and divalent cations, namely, Na^+^, Ca^2+^, and Mg^2+^, while Fe^2+^ and Al^3+^ produced inhibiting effects on flocculating activity. Divalent cations were conclusively demonstrated as the best cation source to enhance flocculation. The bioflocculant works in a wide pH range, from 4.0 to 8.0 with significantly different performances (*P* < 0.05), respectively. It best performs at pH 5.0 and pH 6.0 with flocculating performance of above 90%. A much lower or higher pH would inhibit flocculation. Low dosage requirements were needed for both the cation and bioflocculant, with only an input of 50 mL/L for 0.1% (w/v) CaCl_2_ and 5 mL/L for culture broth, respectively. These results are comparable to other bioflocculants produced by various microorganisms with higher dosage requirements.

## 1. Introduction

Flocculation by a bioflocculant can be described as a process of charge destabilization and bridging induced by polymeric substances produced by microorganisms with the latter playing the more dominant role. Bioflocculants function as an aid in the formation of flocs by suspended particles. These aggregated flocs with their bigger accumulated mass will be more prone to precipitate compared to their smaller original size when in suspension. 

Commonly, usage of commercial flocculating agents such as organic synthetic flocculants: polyacrylamide (PAM) and inorganic macromolecule flocculants: polyaluminium chloride (PAC) had dominated the industry because of their high performance and time saving advantages. However, recent concern on their usage had been identified, namely, as being a highly potential environmental hazard and a health risk to the humans. Aluminium impact to human health has long been disapproved of especially when associated with drinking water supply [1]. Aluminium residues had been reported to cause incidences of Alzheimer's disease while acrylamide poses health concerns from the carcinogenic nature of its monomers besides being nonbiodegradable [2, 3]. Therefore, the usage of microbial flocculants or bioflocculants as alternatives to these commercial organic and inorganic flocculants for water treatment purposes are getting more attention and are being widely recognized worldwide. These include the applications of the bioflocculants in the treatment of raw water such as river water, wastewater treatment [4], and the treatment of drinking water supply [5], for the removal of soil solids, organic and inorganic suspended particles [6], and heavy metals residues [7]. The characteristics of being readily biodegradable and environmentally safe [4], as they are produced naturally, are some of the advantages that make bioflocculants more preferable and acceptable compared to the existing commercial flocculants. 

Various factors have to be considered in determining the optimized performance of a bioflocculant produced by a specific microbe. Cation dependency is one of the essential factors which indicate whether the cation supplied may assist in charge destabilization during the flocculation process by the bioflocculant. Most of the reported bioflocculant-producing microbes such as *Bacillus licheniformis* [8], *Bacillus subtilis* [9], *Bacillus circulans* [5], and the nonbacillus species like *Serratia ficaria *[10] produce bioflocculants that are cation dependent. In comparison, there are only a few reports on cation independent bioflocculants such as *Bacillus *sp. F19 [11] and *Chryseobacterium daeguense* [12]. pH tolerance is another important factor in determining the effectiveness of the bioflocculant in different polluted waters which have wide pH variations [13]. According to Salehizadeh et al. [14], bioflocculation by *Bacillus *sp. As-101 was more prevalent in acidic conditions, while biopolymer flocculant produced by *Bacillus licheniformis *CCRC12826 was effective in neutral pH range [8]. Dosage optimization in water treatment technologies is another aspect to be taken into consideration. It is widely recognized that a lower dosage of bioflocculants with a high performance in flocculating activity will contribute towards cost effectiveness. Furthermore, information on dosage requirement is important for future prospect in water treatment applications. 

 Therefore, the objectives of this study are to determine the cation-dependent characteristics of the bioflocculant produced, to assess the tolerance of the bioflocculant towards pH change, and to establish the dosage requirement of the bioflocculant. This paper will discuss these composite effects on the flocculation performance optimization of a bioflocculant produced by *Bacillus *spp. UPMB13, and the potential of this bioflocculant in water treatment applications.

## 2. Materials and Methods

### 2.1. Bioflocculant Source

A bioflocculant-producing bacterial strain obtained from the culture collection of locally isolated rhizobacteria from oil palm roots, of the Department of Land Management, Universiti of Putra Malaysia identified as *Bacillus *spp. UPMB13 [15] was primarily screened based on its mucoid and ropy colony morphology characteristics as the basic properties for identification of a potential bioflocculant-producing bacterium. Biochemical identification of the strain based on 29 biochemical and enzymatic reaction tests (BBL Crystal Gram-Positive ID System) including hydrolysis of amide and glycosidic bonds with positive release of several fluorescent coumarin derivatives, positive utilization of carbohydrates such as sucrose, mannitol, glycerol, and additional positive utilization of arginine showed high similarity to the characteristics of *Bacillus subtilis* (99%). 

Batch cultures of the strain *Bacillus *spp. UPMB13 were prepared by cultivation in tryptic soy broth on an orbital shaker (150 rpm) at room temperature for the determination of its flocculating capabilities, based on different parameters by kaolin assays [16].

### 2.2. Flocculation Assay

Flocculation assay using kaolin clay as suspended particles was conducted according to the combination of methods described by Feng and Xu [17] and Zheng et al. [11], with modifications. Batch flocculation tests were prepared by suspending 5 g of kaolin clay, with an average size of 4-5 *μ*m, dried at 105°C for 1 hour and kept dry in a dessicator, in 1 L of ultra pure water. The pH of the suspension was then adjusted to 6.8 with HCl or NaOH. After pH adjustment, 50 mL of the suspension was pipetted into 100 mL conical flasks and autoclaved at 121°C for 20 min. The sterile suspensions were subjected to treatments with the addition of 0.5 mL cultured broths (culture aged 24–72 hrs with optical density reading of cell growth at 660 nm varied between 0.5–1.5) and 4.5 mL of 0.1% CaCl_2_. Treatment substitution of sterile ultra pure water and sterile broth acts as control for CaCl_2_ and bioflocculant, respectively. All flasks with respective treatments were transferred onto an orbital shaker and agitated at 200 rpm for 30 s and left to settle for 5 min. The flocculation activity was expressed in two ways. Firstly, by measuring the absorbency (optical density) of the upper phase of the suspensions after settlement using a spectrophotometer at the wavelength of 550 nm.The flocculation activity was calculated according to the following equation by Kurane and Matsuyama [18]:

(1) $$\begin{array}{c} \text{Flocculating}\;\text{activity}\;\left(\%\right)=\;\left[\frac{\left(A-B\right)}{A}\right]\times \;100, \end{array}$$

where *A* is the optical density (OD) of control at 550 nm and *B* is the OD of sample at 550 nm. Secondly, the flocculating activity was determined by visual assessment of the flocs formed by the kaolin particles stimulated by the presence of the bioflocculant.

### 2.3. Cation Dependency

One concern regarding cation dependency in bioflocculation is the effect of different valences of cations on the flocculating activity of the bioflocculant. To investigate this effect, another two treatments (+) Cation and (+) Biofloc were singly added to the basic kaolin assay and acted as controls for comparison to the treatments with the additions of monovalent, divalent, and trivalent cations. Sodium chloride (NaCl) acted as a monovalent cation source; calcium chloride (CaCl_2_), magnesium chloride (MgCl_2_), and iron (II) sulfate (FeSO_4_) acted as the divalent cation source, and aluminium chloride (AlCl_3_) acted as the trivalent cation source. The measurement of flocculating activity was in the same manner as described flocculation assay Section 2.2.

### 2.4. pH Effect

To evaluate the effect of pH on flocculating activity of the bioflocculant, pH of the kaolin suspensions were adjusted to pH 3.0, pH 4.0, pH 5.0, pH 6.0, pH 7.0, pH 8.0, pH 9.0, pH 10.0, and pH 11.0 by the addition of HCl or NaOH accordingly. The purpose of using a wide range of pH measurement is to determine the condition that allows the flocculation process to occur by the aid of the bioflocculant and obtain an optimal range as where it might best performed. The measurement of flocculating activity was in the same manner as described flocculation assay Section 2.2.

### 2.5. Dosage Requirement

The cation source chosen for the determination of cation dosage requirement is CaCl_2_ at a concentration of 0.1% (w/v). Determination of cation dosage is based on percentage from the total volume of kaolin suspension used while the bioflocculant input was kept constant at 1%. From a batch experiment of 50 mL kaolin suspension containing 5 g kaolin/L, the percentage cation dosage chosen was varied with an interval of 5% from 0 to 20%. A subsequent experiment was done based on the result from the first cation dosage experiment to further scrutinize the correct volume of cation to be used.

After confirming the appropriate cation volume to be used, this cation volume was kept constant while the bioflocculant dosage was varied. Determination of bioflocculant dosage was also based on percentage from the total volume of kaolin suspension used. The aim of this dosage requirement was to determine the lowest amount of bioflocculant to be used while achieving the highest flocculating activity. Therefore, the lowest possible dosage chosen was 0.1% and the highest was 2.0%, with an interval of 0.5%. 

The measurement of flocculating activity was in the same manner as described flocculation assay Section 2.2.

### 2.6. Data Analysis

Treatments used for each experiment were done in minimum of three to four replicates with mean and standard deviation values determined. Significant differences were analyzed through analysis of variance (ANOVA), where differences were considered significant at 0.05 confidence level. Prior to the ANOVA test, all data were checked for normally distribution and verified for homogeneity of variances using Levene's test as the basic assumptions for ANOVA. Multiple comparison post-hoc tests, namely, the least significant difference test (LSD) and Games-Howell test were used as the basis for determination of the highest flocculating activity achieved in each assay; for cases of equality of variances assumed or not assumed, respectively. 

## 3. Results and Discussion

### 3.1. Cation Dependency of the Bioflocculant Produced by UPMB13

**Figure 1:**
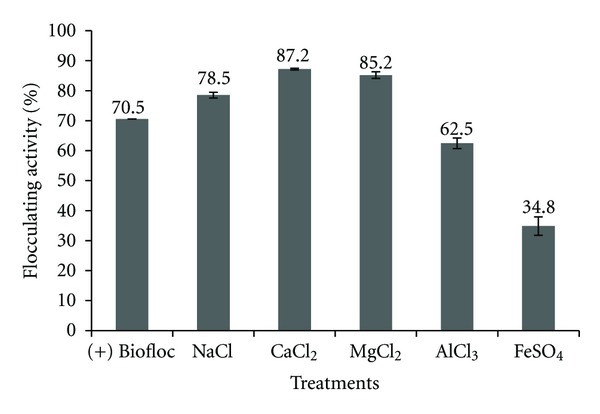
The effect of monovalent, divalent, and trivalent cations on the flocculating activity of bioflocculant produced by *Bacillus *spp. UPMB13. (+) Biofloc treatment act as control for comparison with the other cation treatments.

Effect of different sources of cation on flocculating activity of bioflocculant produced by UPMB13 was evaluated and is depicted in Figure 1. Flocculating activity of UPMB13 bioflocculant on kaolin suspension was significantly affected with the addition of cations. Synergistic effects were observed with the addition of Na^+^, Ca^2+^, and Mg^2+^ while the addition of Al^3+^ and Fe^2+^ leads to a decrease in flocculating activity both in terms of optical density percentage and observation of flocs formation. 

The principle of cation addition in bioflocculation is basically for the purpose of charge destabilization of negative repulsion charges of the suspended particles and the residual charge of functional groups of the bioflocculant [14]. This is true according to the study by Salehizadeh and Shojaosadati [19], whereby the cations used decrease the negative electrical charge of kaolin particles and the biopolymer flocculant. Cation acts as a coagulant which neutralizes the zeta potential in the kaolin suspension allowing the suspended kaolin particles to be attracted and bind together before the bridging mechanism of the bioflocculant occurs.

Addition of monovalent cation, namely, Na^+^ has a slight synergistic effect of about 8% on flocculating activity. Monovalent cations produce bonds that are loose in structure and, therefore, result in a decrease in floc density, size, and floc resistency to shear compared to divalent cations [9].

Al^3+^ acts as a trivalent cation source. From the result, the flocculating activity based on optical density for the treatment with the addition of Al^3+^ is 62.5% indicating that the upper phase of the kaolin suspension is quite clear. However, this result is still lower than the (+) Biofloc treatment, without any addition of cations. Furthermore, there was no floc formation observed in the suspension. This suggests that the clarity of the suspension was not due to the mechanism of the bioflocculant treatment. A comparison of this result to a control treatment containing only the cation, without any addition of bioflocculant, confirmed this finding whereby a similar optical density reading was observed (data not included). Further investigation was done by subsequently measuring the pH of the resulting suspension (data not included). It was found that the pH for the treatment with Al^3+^ was slightly acidic with a pH range of 4.19 to 5.76 for the four replicates. Therefore the pH effect is suggested to be the reason which inhibits bioflocculant activity while the clear phase was only due to the destabilization of charges by the trivalent cation. This is in accordance with Shih et al. [8], where a dramatic decrease of flocculating activity of *Bacillus licheniformis* CCRC with the addition of Al^3+^ was due to the drop in pH. Additionally, Gong et al. [10] reported that trivalent cations could change the surface charge of kaolin particles and cover the adsorb sites which lead to low flocculating activity. Moreover, the presence of aluminium ion itself might be the inhibitory factor as it is known to give rise to environmental problems [5] thus, making the suspension unfavorable for most microbial activities to occur.

Divalent cations, namely, Ca^2+^ and Mg^2+^ were proven to be the best cation source to aid flocculation by UPMB13 bioflocculant with flocculating activity of 85% and above, a significant increase (*P* < 0.05) of about 15% from control (+) Biofloc treatment (Table 1). Similar findings were also reported by Salehizadeh and Shojaosadati [19] and by Gong et al. [10], where apparently divalent cations such as Ca^2+^ and Mg^2+^ have the strongest stimulating effect and were more effective compared to monovalent or trivalent cations. However, this is not true for Fe^2+^ where the treatment does not induce flocs formation and the optical density reading does not even reflect the mechanism of charge destabilization by a divalent cation when compared to the control treatment of only Fe^2+^ ion (data not included). This suggests that the sole presence of Fe^2+^ ion inhibits flocculation process by the bioflocculant. Similar findings were reported by Takeda et al. [20] and Wu and Ye [9] whereby they concluded that excessive supply of Fe^3+^ and Al^3+^ ions will inhibit flocculation due to excessive adsorption of the ions.

### 3.2. pH Tolerance of the Bioflocculant Produced by UPMB13

**Figure 2:**
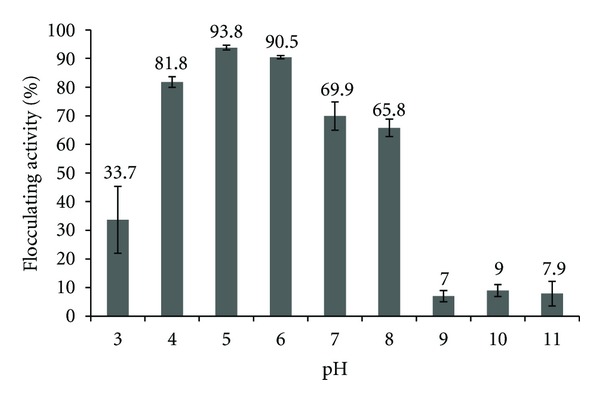
The effect of acidic and alkaline conditions on flocculating activity of bioflocculant produced by *Bacillus *spp. UPMB13. The pH range selected was between pH 3.0–11.0.

Bioflocculant produced by UPMB13 has a relatively wide pH tolerance ranging from slightly acidic to slightly alkaline conditions (Figure 2). The result shows that the bioflocculant can perform at pH ranges from 4.0–8.0, though, with significantly different performance (*P* < 0.05) (Table 2). The highest flocculating activity is achieved at pH 5.0 with a mean difference of about 86% from the lowest activity at pH 9.0, 10.0, and 11.0 (*P* > 0.05). The bioflocculant performance in percentage was found to be similar for pH 4.0, 7.0, and 8.0 (*P* > 0.05); however, it is noted that more flocs are formed in the treatment at pH 4 compared with the other two pH. At pH 6.0, the flocculating activity was found to be significantly 3.3% lower than pH 5.0 (*P* < 0.05) though in both conditions the bioflocculant performance reached above 90% activity.

Similar finding was also reported by Liu et al. [12] for bioflocculant produced by *Chryseobacterium daeguense* W6 which preferred conditions of low acidic to low alkaline with the same pH range of 4.0–8.0.

In highly acidic condition of pH 3.0, the flocculating activity (optical density) was found to be 33.7%. Although the upper phase of the treated suspension was clear, it is noted that there were no flocs formation observed implying that the clear upper phase was not due to the flocculation mechanism of the bioflocculant, but rather it was due to the natural impact of acidity on suspended particles through charge destabilization. On the other hand, in alkaline conditions of pH 9.0, 10.0, and 11.0, declination of flocculating activity occurred as the flocculating activity drastically decreased but with very small flocs slightly present. This maybe due to the restabilization of the kaolin particles which inhibit agglomeration and bridging by the bioflocculant. This is true according to Wang et al. [13] whereby they stated that pH may determine the formation of flocs as well as affect the stability of suspended particles.

pH tolerance by a bioflocculant usually does not reflect the optimum pH for the growth of its producer [21] or the optimum pH of its production, rather it may lie within a wider pH range or it may lie outside the range of what is preferred for growth and production or basically is totally opposite to them. Zheng et al. [11] stated that *Bacillus *sp. F19 growth was not affected by any pH changes, but the production of bioflocculant MBFF10 by the strain proliferates at high pH of around 7.0–12.0. In contrast, the best pH for flocculating activity to occur was found to be at pH 2.0 and a decrement in flocculating activity could be seen from acidic to alkaline conditions. Another example is on a bioflocculant produced by *Citrobacter *sp. TKF04 discussed by Fujita et al. [6] where they found that the best pH for the bacterium growth was within the range of pH 7.2 to pH 10.0 while for effective flocculation the pH range is from pH 2.0 to pH 6.0.

In comparison, bioflocculant produced by UPMB13 does reflect the optimum pH of 6.8 for growth of its producer and its production. However, the flocculating activity vary within a range around the optimal value, from 4.0–8.0. It is noted that this data applies only to kaolin suspensions as pH tolerance of this bioflocculant may vary with other solid suspensions; therefore further pH assays are needed. 

### 3.3. Dosage Requirement

#### 3.3.1. Cation Dosage

**Figure 3:**
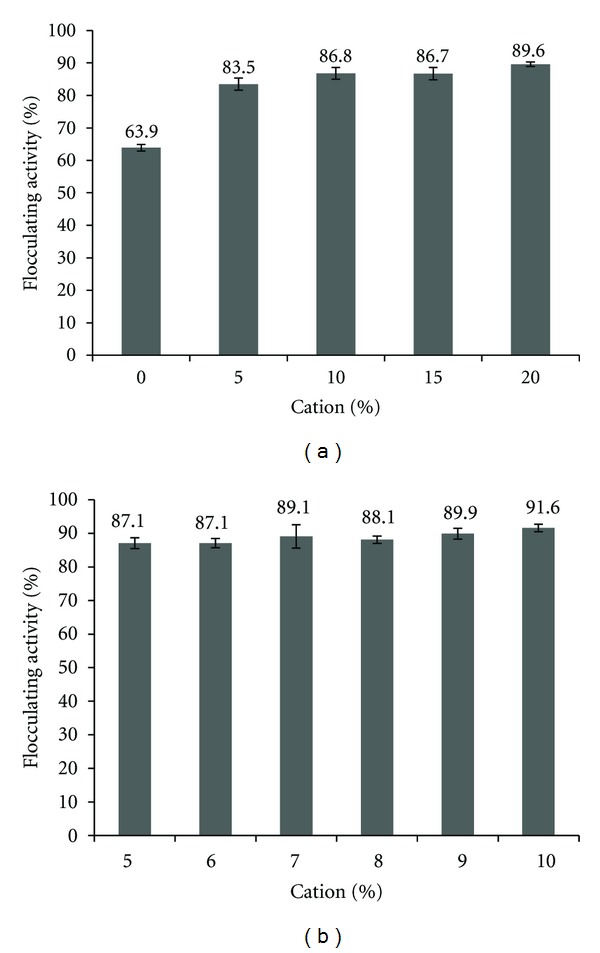
(a) Flocculating activity at different cation dosage as percentile of total volume of suspension (0–20%). (b) Flocculating activity at different cation dosage as percentile of total volume of suspension (5–10%).

As the bioflocculant produced by UPMB13 is cation dependent, it is important to determine the optimal cation dosage which will not overcome the positive effect of the bioflocculant. The cation dosage requirement is as described in Figure 3. It was found that the highest amount of cation to be supplied for optimal flocculation, based on the specific UPMB13 batch culture used, as percentile of total volume, was at 10% as it was the lowest dosage input with high flocculating activity of 86.8% (Figure 3(a)). This was chosen based on the statistical analysis where there are no significant differences (*P* > 0.05) between 10–20% cation input (Table 3).

To further determine the correct amount of cation to be used which will not waste the cation source or over supply the cation dosage above what should already be sufficient, a subsequent test between 5–10% dosage input was carried out (Figure 3(b)) resulting to the conclusion that 5% volume input was the optimal dosage with no significant difference (*P* > 0.05) observed with each increment of 1% input up to 10%.

#### 3.3.2. Bioflocculant Dosage

**Figure 4:**
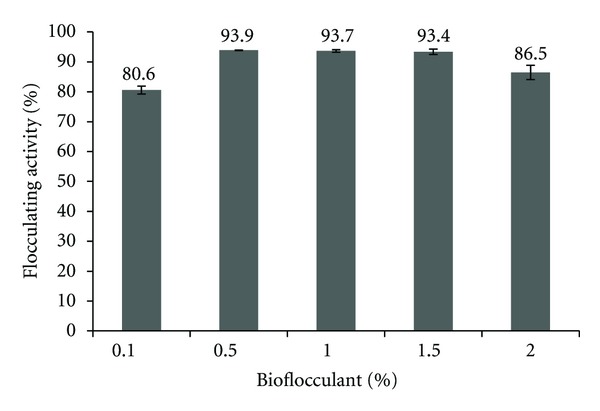
Flocculating activity based on different bioflocculant dosage supplied with the range of 0.1–2.0% from total volume of suspension.

 According to Gong et al. [10] inadequate dosage of bioflocculant will lead to a poor bridging phenomenon, thus resulting in low flocculating activity while excess input might induce re-stabilization of kaolin particles. The result obtained is in accordance with this reported findings whereby the lowest (0.1%) amount of bioflocculant may only reach to about 81% and the highest (2.0%) produced a drop in flocculating activity to 87% while the optimal dosage was between 0.5–1.5% (*P* > 0.05) with 94% flocculating activity (Figure 4). It is noted that the result is specific to the batch culture used in the experiment whereas the maximum and minimum flocculating activities of other batch cultures might vary with each experiment conducted. However, in terms of percentage volume, the finding may be acceptably applied for all conditions. Thus, 0.5% (5 mL/L) bioflocculant dosage input is considered the best and adequate volume to be used (Table 4). Bioflocculant produced by UPMB13 was proven to be an effective flocculant with high flocculating activity achieved at a low dosage input. 

 Summary of differences between various bioflocculant-producing microorganisms based on bioflocculant dosage, cation dependence and pH tolerance are depicted in Table 5. In terms of cation dependency, it can be seen that most bioflocculant-producing microorganisms produces cation dependent bioflocculant with exception of *Citrobacter *sp. TKF04 [6]. However, the effect of different types of cation sources is subjective to each organism. One common trait between UPMB13 and the other reported microorganisms is the positive influence of Ca^2+^ ions in aiding flocculation. pH tolerance of the bioflocculant produced by UPMB13 is noticeably wider compared with others, similar to that of *Chryseobacterium daeguense* W6 [12] and undeniably less than that of *Bacillus circulans* X3 [5], at which the strain is more alkaline tolerant. Nevertheless, it is highly noted that the dosage requirement of UPMB13 bioflocculant is much lower comparable to the others [6, 8, 14, 22]. 

## 4. Conclusions

Flocculation performance optimization of a bioflocculant produced by *Bacillus *spp. UPMB13 through kaolin assays was investigated. Positive synergistic effects can be seen with the addition of NaCl, CaCl_2_, and MgCl_2_ at which these cation sources are cheap and readily available. The bioflocculant has a wide pH tolerance and is capable to perform in the pH range of 4.0–8.0, away from neutral pH range (7.0 + 2.0). Bioflocculant produced by UPMB13 has low dosage requirement of 5 mL/L culture broth, comparable with other reported bioflocculants. Thereby, UPMB13 bioflocculant is considered as a potential bioflocculant with low dosage requirement and wide pH tolerance and may be assisted with cheap cation sources for future prospect in suspended solids' pollution treatment in wastewater, river water, and drinking water applications.

## Figures and Tables

**Table 1:** Statistical analysis for flocculating activity with different cation treatments added with UPMB13 bioflocculant.

Cations	Flocculating activity (%)	*P* value*	Homogenous subset**
NaCl	78.5 ± 1.95	*P* ≤ 0.05	c
CaCl_2_	87.2 ± 0.53	*P* > 0.05	d
MgCl_2_	85.2 ± 2.19	*P* > 0.05	d
AlCl_3_	62.5 ± 3.55	*P* ≤ 0.05	b
FeSO_4_	34.8 ± 6.20	*P* ≤ 0.05	a

^∗^The mean difference between groups is significant at 0.05 level.

^∗∗^Homogenous subset is in ascending manner from lowest to the highest mean value.

**Table 2:** Statistical analysis for flocculating activity at different pH conditions.

pH	Flocculating activity (%)	*F* value	*P* value*
3.0	33.7 ± 11.6	242.398	.000
4.0	81.8 ± 1.8		
5.0	93.8 ± 0.9		
6.0	90.5 ± 0.5		
7.0	69.9 ± 4.9		
8.0	65.8 ± 3.1		
9.0	7.0 ± 2.0		
10.0	9.0 ± 2.1		
11.0	7.9 ± 4.3		

Equality of variances not assumed. Games-Howell post-hoc test was used.

*The mean difference between groups is significant at 0.05 level.

**Table 3:** Statistical analysis for flocculating activity at 0–20% cation dosage.

Dosage (%)	Flocculating activity (%)	*P* value*	Homogenous subset**
0%	63.9 ± 0.98	*P* ≤ 0.05	a
5%	83.5 ± 1.86	*P* ≤ 0.05	b
10%	86.8 ± 1.81	*P* > 0.05	c
15%	86.7 ± 1.91	*P* > 0.05	c
20%	89.6 ±0.70	*P* > 0.05	c

*The mean difference between groups is significant at 0.05 level.

**Homogenous subset is in ascending manner from lowest to the highest mean value.

**Table 4:** Statistical analysis for flocculating activity at 0.1–2.0% bioflocculant dosage.

Dosage (%)	Flocculating activity (%)	*P* value*	Homogenous subset**
0.1%	79.8 ± 1.65	*P* ≤ 0.05	a
0.5%	94.2 ± 0.44	*P* > 0.05	c
1.0%	93.2 ± 1.01	*P* > 0.05	c
1.5%	93.6 ± 0.72	*P* > 0.05	c
2.0%	86.6 ± 1.78	*P* ≤ 0.05	b

*The mean difference between groups is significant at 0.05 level.

**Homogenous subset is in ascending manner from lowest to the highest mean value.

**Table 5:** Bioflocculant dosage, cation dependence and pH tolerance of different bioflocculant-producing microorganisms.

Microorganism	Dosage (mL/L)	Cations	pH	Reference
*Citrobacter *sp. TKF04	100	No ions	2.0–6.0	[6]
*Vagococcus'* sp. W31	10	Ca2^+^	7.0–10.0	[22]
*Bacillus circulans* X3	2^a^	Ca2^+^, Fe3^+^, Al3^+^	4.0–10.0	[5]
*Chryseobacterium daeguense* W6	1.2^a^	K^+^, Ca2^+^, Mg2^+^, Mn2^+^	4.0–8.0	[12]
*Bacillus coagulants* As101	40	Ca2^+^, Fe3^+^, Al3^+^	3.7	[14]
*Bacillus licheniformis* CCRC 12826	150	Ca2^+^, Fe3^+^, Al3^+^	6.4–7.1	[8]
*Bacillus subtilis* DYU1	40^a^	Ca2^+^, Mg2^+^	6.0–7.0	[9]
*Bacillus *spp. UPMB13	5	Na^+^, Ca2^+^, Mg2^+^	4.0–8.0	Present article

^a^The dosage of the bioflocculant used was expressed in mg/L.
